# Nanocluster-Based Drug Delivery and Theranostic Systems: Towards Cancer Therapy

**DOI:** 10.3390/polym14061188

**Published:** 2022-03-16

**Authors:** Alma Lucia Villela Zumaya, Rosica Mincheva, Jean-Marie Raquez, Fatima Hassouna

**Affiliations:** 1 Faculty of Chemical Engineering, University of Chemistry and Technology Prague, 166 28 Prague, Czech Republic; zumayaa@vscht.cz; 2 Laboratory of Polymeric and Composite Materials, University of Mons (UMONS), Place du Parc 20, 7000 Mons, Belgium; rosica.mincheva@umons.ac.be (R.M.); jean-marie.raquez@umons.ac.be (J.-M.R.)

**Keywords:** nanoclusters, inorganic nanoparticles, polymeric nanoparticles, theranostics, drug delivery, cancer therapy

## Abstract

Over the last decades, the global life expectancy of the population has increased, and so, consequently, has the risk of cancer development. Despite the improvement in cancer therapies (e.g., drug delivery systems (DDS) and theranostics), in many cases recurrence continues to be a challenging issue. In this matter, the development of nanotechnology has led to an array of possibilities for cancer treatment. One of the most promising therapies focuses on the assembly of hierarchical structures in the form of nanoclusters, as this approach involves preparing individual building blocks while avoiding handling toxic chemicals in the presence of biomolecules. This review aims at presenting an overview of the major advances made in developing nanoclusters based on polymeric nanoparticles (PNPs) and/or inorganic NPs. The preparation methods and the features of the NPs used in the construction of the nanoclusters were described. Afterwards, the design, fabrication and properties of the two main classes of nanoclusters, namely noble-metal nanoclusters and hybrid (i.e., hetero) nanoclusters and their mode of action in cancer therapy, were summarized.

## 1. Introduction

Cancer is one of the main causes of death around the world, with 19.3 million cases being diagnosed worldwide in 2020 according to World Health Organization [1]. It is estimated that there will be 27.5 million new cases of cancer each year by 2040 [2]. Over the years, conventional treatments such as radiation and chemotherapy have improved greatly, and yet drawbacks and challenges encountered during treatment are still present, such as cytotoxicity, non-specific distribution of the anticancer agents, low concentration of the drug reaching the target tumor site and the development of multiple drug resistance [3]. In the last decades, the understanding of the human body has led to a great range of possibilities for cancer treatment [4]. For example, the emergence of nanotechnology, as a discipline of science for the development of tools and devices with sizes of 5 to 300 nm, has led it to become a key component in nanomedicine for the preparation of therapy and drug delivery agents, which have been useful over the last few decades [5]. The advantages of nanomedicine consist of materials that are designed at the nanoscale level with the ability to exhibit novel properties compared to their bulk counterpart. These nanostructures are also capable of encapsulating or attaching drugs for the delivery to the target tissues, as they can increase the bioavailability and reduce the dose, toxicity and transport across biological barriers [6]. The application of these nanostructures can range from theranostics (combination of therapy and diagnostics), tissue engineering, drug delivery, targeted therapy, and imaging [7]. The adoption of nanocarriers between 10–100 nm as a cancer treatment has reached clinical trials, showing their advantage over conventional treatments and their improved efficiency [8]. They have abilities associated with improved biodistribution and pharmacokinetics, not to mention the increased solubility of hydrophobic compounds. Thanks to their unique properties, they can be designed as systems with improved efficacy and reduced side effects [9]. Despite their advantages, single DDS are usually not sufficient to prevent cancer recurrence; hence, the possibility to apply simultaneous therapeutic agents would substantially increase the efficacy of the treatment. The ability to incorporate different components with multiple mechanisms of action against cancer cells has been proposed since the 1960s as an alternative to tackle multiple drug resistance. The first use of combination therapy was developed as a pilot study in 1963 by Moxley et al. [10] against Hodgkin’s lymphoma. Methodologies combining various functionalities within a single particle with promising properties have been reported in the literature [11,12,13,14,15]. However, despite the attractive features of this approach, it often involves the usage of toxic chemicals that come into contact with the biological molecules, which could cause bioactivity loss. In this respect, assembly of different building blocks, which can be prepared individually while avoiding handling of toxic substances when manipulating biological materials, into large hierarchical structures in the form of nanoclusters, would be more advantageous. A plethora of building blocks can be applied in the preparation of the nanoclusters for cancer therapy. The search for nanocarriers with low toxicity, better biocompatibility and biodistribution has gained more attention, comprising biodegradable polymers and inorganic NPs. This review focuses on this concept of the assembly of hierarchical structures in the form of nanoclusters as DDS and theranostics agents, the major features of the building blocks that can be employed for their preparation, the mechanisms responsible for their formation and their drug delivery process. Emphasis on biodegradable and noble-metal NPs based on gold, silver and iron oxide for the assembly of the nanoclusters is discussed in this review.

## 2. Nano-Based Building Blocks

In current cancer therapy, the number of designed and tested nanocarriers continuously increases and includes micelles, dendrimers, liposomes, vesicles, capsules, organic and inorganic NPs, etc. [16,17,18,19,20]. An ideal nanocarrier for drug delivery and cancer treatment should meet a number of criteria including avoidance of the cleansing by the reticuloendothelial system (RES) and release and accumulation of the cargo at the targeted site with the desired therapeutic dose [21]. These criteria are directly dependent upon the particle size, surface chemistry and charge, as well as hydrophobicity. Among these, particle size distribution, surface charge and surface chemistry are important for successful design of the nanoclusters with controlled size, morphology and stability. Therefore, rational design of the nanoclusters using viable strategies requires a design of the building blocks with tailored properties. 

### 2.1. Polymeric Nanocarriers as Building Blocks

Targeting the ideal nanocarrier for cancer imaging and treatment, scientists have focused much effort on PNPs. Indeed, PNPs have already been recognized as good candidates for combined treatment and diagnosis of cancer. This is based on PNPs’ remarkable stability, biocompatibility and/or biodegradability, design possibilities and relatively low cost. Case-dependent application of basic chemistry in the design, the synthesis and the modification of polymers allows the design of pre-defined materials selectively, combining different elements in the so called “lego” concept towards cancer imaging and therapy [22].

The large number and the huge versatility of synthetic/modification processes for polymers allow precise control over chemistry, functionality, architecture and self-assembly. Selective conditions provide loading of anti-cancer substances, and surface “decoration” with different reactive moieties allows the overcoming of problems related to immunogenicity, circulation time, cargo release, targeted delivery, traceability, and synergistic chemotherapy [4,23,24,25,26,27,28,29]. Using this panoply of possibilities, PNPs can be designed to accumulate in tumor sites passively or actively, respond to particular tumor microenvironment conditions (pH, reactive oxygen species, overexpressed enzymes) and trigger and control drug/imaging agent delivery and exposure to provide the appropriate therapeutic or imaging outcome of a theranostic.

As an example, multiple types of polymers have been designed to form micelles, vesicles, nanospheres, nanogels, etc., and have been used in cancer diagnostics, imaging and/therapy (Table 1). Although biocompatible, these polymers usually face size, surface charge, stability and blood-residence time restrictions, predetermining the in vivo fate of the PNPs [30]. Indeed, the lack of biodegradability of polyethers raises significant concerns for bioaccumulation at the targeted body sites after drug-delivery is achieved. This requires a clause for elimination through the natural pathways (preferably renal filtration but also gastro-intestinal) to be fulfilled prior to application in cancer-targeting formulations. As renal filtration is only possible below a certain size, the molecular weight to be respected lies preferably below 5 kilodaltons (kDa) for a polyethylene glycol (PEG)-containing nanocarriers, for example [31,32,33]. Thus, depending on their size and chemistry, PNPs will distribute and accumulate either in the kidneys, liver or spleen, with much less concentration in other organs and tissues [34]. Actually, it is all a question of compromise—the desired bioaccumulation at the targeted size vs. bioelimination after drug-delivery must both be optimal. A very good example is related to PEG-based systems for tumor targeting. Because of the particularities of the tumor tissue, namely vasculature, PEG nanocarriers designed for photodynamic therapy (PDT) show tumor accumulation, which increases with the molecular weight of the carrier (10 < 20 < 30 ≪ 40 ≪ 60 kDa) [7], while for successful bioelimination a molecular weight below 5 kDa is required. The problems related to the elimination of non-biodegradable polymers might be solved by the use of biodegradable polymers, such as (but not limited to) hydroxypropyl (methyl)cellulose), carbomers, sodium hyaluronate, chitosan (CHIT), cyclodextrins, poly (galacturonic acid), xyloglucan, xanthan gum, gellan gum, poly (ortho esters), poly (glycolide) (PGA), poly (lactide) (PLA), poly (caprolactone) (PCL) and poly (lactide-co-glycolide) (PLGA). These synthetic or natural polymers are recognized by living organs, tissues and cells in vivo, and enter the complex metabolic pathways catalyzed by enzymes to finally be converted to energy, building blocks or water and carbon dioxide (CO_2_). 

The typical PNPs might be defined as reversibly formed assemblies (micelles, liposomes, dendrimer systems or solid NPs) of multiple polymer chains, in which active substances are encapsulated, mixed, absorbed or attached [48,49,50,51,52]. Their bulk is usually charged with therapeutic and/or imaging agents or diagnostics, such as medications, gold (Au) or iron oxide (Fe_3_O_4_) NPs, etc., and their surface was decorated with peptides [53], ribonucleic acid (RNA) [54], folic acid (FA) [55], boronopicolinic acid [56] and other biologically active functionalities. Some authors, ref. [57], designed acid-responsive copolymers (named Dlinkm) and micelle-based systems from (Micelleplex = PEG-Dlinkm-R9-PCL) by interacting with small interfering RNA (siRNA). These Micelleplex are obtained via the solvent exchange method. The in vivo tests on nude mice have shown improved siRNA protection towards serum and prolonged blood-circulation time, as well as improved anticancer activity when Dlinkm copolymer was present in micelles structure. Another group [58] loaded poly (lactic-co-glycolic acid)-co-polyspermine (PLGA-PSPE) micelles with dihydroergotamine (DHE) and studied DHE uptake by A549 lung cancer cell lines together with the in vivo cytotoxicity in lung tumor model mice. These micelles showed controlled and sustained release of the loaded drug and dose-dependent cell-uptake. Enhanced apoptosis was attested by a cell viability study. Reduced cancer cell number and size were also observed for micelles in comparison to free drug. Polymeric micelles were found not only to provide improved anticancer activity but also decreased medication side-effects (namely cardiotoxicity) in the case of acetylthevetin B (ATB)-loaded CHIT/pluronic P123 micelles [59].

The building and loading versatility of the polymeric micelles allowed further development of the so called “co-delivery strategy”—an effective method to minimize the amount of needed medication by amplifying its effect via a synergy with other biologically active substances. This was firstly demonstrated [60] in a pH-sensitive CHIT/pluronic F127 micelles with conjugated doxorubicin (DOX) and physically loaded paclitaxel in the hydrophobic core of the micelles. Interestingly, the loading capacity of the micelles towards paclitaxel was found to increase with DOX-conjugate content. The release of the active substances followed in vivo a four-fold increase of the plasma concentration time compared to mixed paclitaxel- DOX non-micelle formulations. A similar example is the DOX-conjugated methoxypoly (ethylene glycol)-poly (caprolactone) (mPEG-PCL) diblock copolymer self-assembled into micelles in a curcumin aqueous solution. These co-delivery micelles proved in vitro cytotoxic activity against A549 tumor cells greater than the free medications [60].

**Figure 1:**
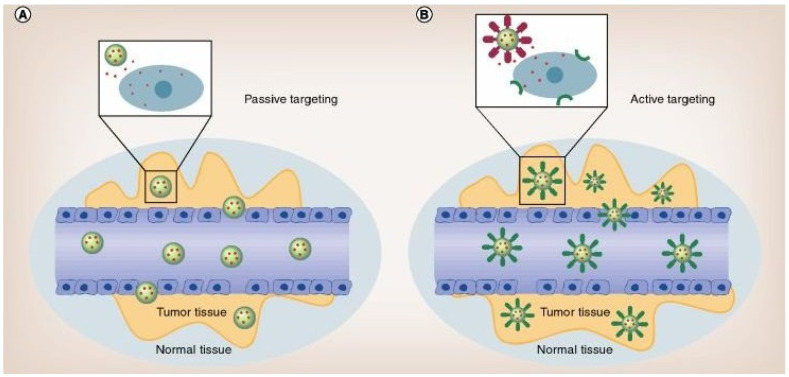
Schematic of passive (**A**) vs. active (**B**) targeting in cancer diagnostic, imaging and therapy. Reproduced with permission from ref. [61]; Boateng et al., Int. J. Mol. Sci. 21 (2020) 273. (open access Creative Common CC).

Despite all benefits, the concept of building PNPs via self-assembly in the presence of an active substance, a metallic nanoparticle or other biologically active moiety presents some disadvantages. Thus, the PNPs can be regarded as a trap controlling the accessibility and limiting the action of any anticancer agent. The success of the as obtained PNPs is controlled by the particle size, shape and surface potential. Additionally, their interaction with tumors solely occurs via a passive targeting (a diffusion-controlled, and thus size-controlled, migration of a substance through the blood vessels endothelium with no structural change or specific interactions (Figure 1) [61]) allowed by the increased permeability and retention (EPR) effect related to the leaky tumor vasculature [62,63]. As this passive targeting is often non-specific and inefficient, the PNPs’ efficiency is substantially limited [64].

Another introduced concept utilizes the so-called active targeting (Figure 1), e.g., specifically introduced surface chemistry of preformed PNPs as an anchoring site for the selective introduction of functional groups (antigens or antibodies (Ab)), stealth chains (PEG brushes), traceable markers (fluorescent probes) and synergistic chemotherapy (drugs and metal clusters) targeting corresponding functionalities on the tumor cell membrane. Gifted with tumor specificity, these surface modified PNPs present active tumor targeting through binding with tumor-specific receptors [65,66,67]. As an example, phenylboronic acid-decorated chondroitin sulfate A (CSA)-based PNPs with a dual-targeting function were developed [50]. Folate-bearing nanotheranostics were also obtained using sulfur-containing hyperbranched polymer [68]. Other exemplary studies are summarized and discussed in a recent review [69]. Despite this, conjugation of functional moieties and tags to PNPs demands precise chemical design and control over synthesis, as well as functionalization via differing chemistries and via covalent bonding that might also affect the integrity of the PNPs and the trapped medicine [70,71,72,73,74].

Originally, other author groups benefitted from the highly selective, strong but dynamic host-guest (supramolecular, non-covalent) interactions in the design and construction of active nanoparticles for cancer treatment [22,75,76,77,78]. For instance, the strong interactions between cucurbit[n]urils (where n = 5–8, or 10) and spermine were employed in developing PNPs for combined cancer imaging and chemotherapy [62,63,64,65]. Sun et al. [22] used PLA/PLGA PNPs as independent “lego” blocks to decorate with cucurbit[7]uril. A second complementary “lego” piece (amantadine, amantadine conjugated FA, PEG and fluorescein isothiocyanate) was also linked with and allowed the incorporation of a second drug (oxaliplatin), in addition to the first loaded drug (paclitaxel), for a possible synergistic chemotherapy.

**Figure 2:**
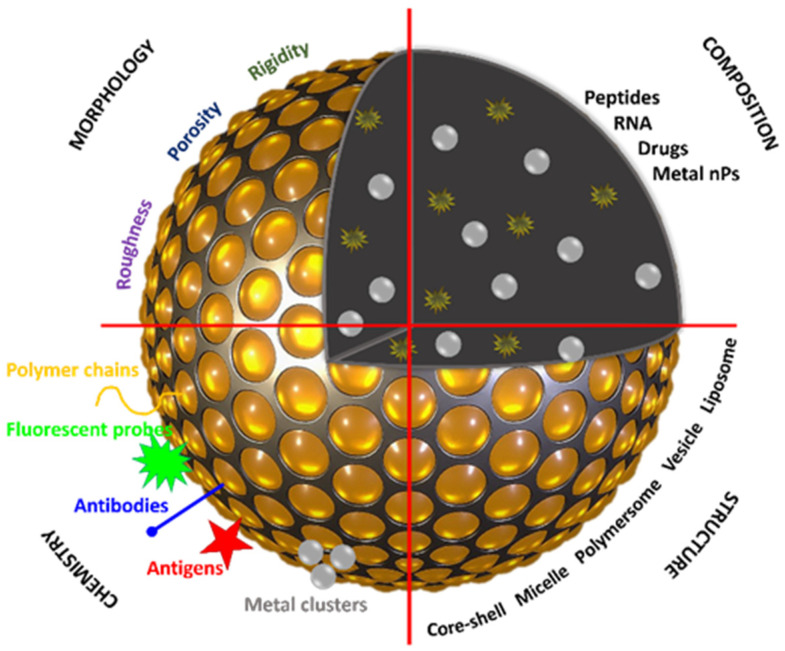
Schematic of the versatility of PNPs in cancer diagnostic, imaging and therapy.

Based on the above, the PNPs might be presented using the concept of multifunctional “lego” particles, which structure, composition, morphology and surface can be on-demand adapted to act in diagnostics, imaging and even treatment of cancer [22] (Figure 2). To conclude, the structural, physical and chemical versatility and tenability of the polymeric nanocarriers, which are capable of loading various types of anticancer therapeutic agents, constitute an interesting building block platform for the construction of nanoclusters-based DDS.

### 2.2. Inorganic Nanoparticles

The design and fabrication of numerous ranges of DDS with the ability to tailor the composition, size and functionality have provided immense resources in cancer therapy and can be part of the assembly of the nanoclusters as DDS. Inorganic NPs made of gold, silver, iron oxide, platinum, copper and so on have been used more frequently in nanomedicine thanks to their size and material dependent physico-chemical properties. In particular, their optical and magnetic properties, ease of functionalization and inertness allow them to be a good alternative for imaging and ablation of cancer cells [79]. This review focuses on the most investigated inorganic NPs and their related nanoclusters for cancer therapy, namely gold, silver and iron oxide.

#### 2.2.1. Gold Nanoparticles

The attention to gold (Au) in DDS and in theranostics agents has a long history thanks to its chemical stability, ease of synthesis, non-immunogenic and low toxicity. When the size of Au is on the nanometer scale (i.e., 1–100 nm), it displays totally different properties from bulk Au, particularly its optical properties. Au NPs offer unique properties, including a surface plasmon resonance (SPR) effect, which possesses a high light-to-heat conversion efficiency of due to the oscillating free electrons in their conduction bands, size- and shape-dependent electronic properties and photothermal effect, thus attracting remarkable attention and practical consideration over the last decades. Their biomedical applications include drug and gene delivery, photothermal therapy (PTT), photodynamic therapy, radiotherapy and use as contrast agents for cancer imaging [80]. In particular, the recent advances in the engineering of Au NPs allowed a better control and tuning of the size, shape, composition and surface chemistry of the particles and thereby adjustment of the optical and electronic properties of Au NPs for the effective utilization of these materials in biomedical applications [81,82,83]. The synthesis of Au NPs was first reported in 1941 upon reacting with chloroauric acid (HAuCl_4_) and trisodium citrate (Na_3_C_6_H_5_O_7_) [84]. In 1951, Turkevich et al. reported in detail what has become since then the most common method of Au NPs synthesis. This method is also based on the reduction of HAuCl_4_ using Na_3_C_6_H_5_O_7_ as a reducing agent as well as a surface ligand on the surface of Au NPs [85]. Adjustment of this method allowed the preparation of spherical monodisperse Au NPs in the size range between 15 to 150 nm, depending on the initial concentration of Na_3_C_6_H_5_O_7_. This methodology was the foundation for the development of other techniques that allowed a more controlled synthesis of Au NPs in diverse media (e.g., water, organic solvents) at different pH, temperature and reducing agents (e.g., sodium borohydrate (NaBH_4_) [86,87] and aspartate [88]). The Brust-Schiffrin method is another approach used to synthesize Au NPs in organic solvents that are not miscible with water. The methodology involves the transfer of gold(III) chloride (AuCl_4_) to an organic solvent (e.g., toluene, chloroform, benzene) phase from an aqueous solution using tetraoctylammonium bromide (TOAB) as the phase-transfer agent, and reduced by NaBH_4_, in the presence of an alkanethiol (e.g., dodecanethiol) [87]. Other reported methods of Au NPs synthesis include electrochemical, seeding growth, biological, microwave irradiation and sonochemical. The variety of methods of synthesis allowed the production of Au NPs with multiple shapes (e.g., nanorods, nanocubes, nanocages and nanotubes) and size and surface chemistry, thus allowing precise control of their properties [89,90,91,92,93]. Beyond this, the possibility to bind amines, thiols and polymers to the surface of Au NPs offers a suitable way to incorporate reactive functional groups that can be used for conjugating therapeutic agents (e.g., drugs, siRNA, radionuclides photosensitizers and genes) and for targeting (e.g., peptides and Ab). 

This makes Au NPs a promising material in the development of polyvalent nanomedicines capable of multimodal therapeutic applications in cancer treatment [94,95]. In general, surface functionalization can be achieved through covalent attachment (e.g., thiol linkages) or physical adsorption. PEGylation is one of the ubiquitously used strategies for surface functionalization of Au NPs [96,97]. PEG is versatile and inexpensive, and it is Food and Drug Administration (FDA) approved. It is commercially available in different molecular weights and with various functional groups such as thiols, acids, amines or even vitamins, enzymes, etc. PEG can be attached covalently to the Au NPs surface. PEGylated Au NPs are characterized by reduced uptake by the RES, decreased enzymatic degradation, diminished renal filtration and prolonged half-blood life due to an increase in their hydrophilicity, and hence an enhanced bioavailability [98,99]. All these properties are present because PEGylation prevents the formation of a protein corona, so that the immune system does not recognize the Au NPs. The last, but not the least, PEGylation allows minimizing nonspecific interactions via steric stabilization and surface charge control to avoid NP loss to unwanted locations [100]. Despite these advantages, recent in vivo studies showed that PEGylation may induce acute inflammation and apoptosis in liver cells in the presence of PEGylated Au nanoshells (AuNSs) [101,102]. When it comes to the nanometer scale, Au NPs exhibit various advantages compared to their bulk counterpart, and their numerous applications in the biomedical area has led to the exploration of their in vitro and in vivo adverse effects [103,104]. In fact, it was shown that Au NPs can exhibit toxicity, which is dependent on their different properties such as size, shape, coating material and surface charge [105,106]. Nonetheless, it is possible, with proper surface modifications, to reduce or even eliminate their toxic effect and still use them as therapeutic agents. Besides PEG, different surface modifications were studied to reduce or eliminate the cytotoxic effect of Au NPs such as polyacrylamide [107], polyvinylpyrrolidone (PVP) [108], oligonucleotides [109], carbohydrates [110], folic acid (FA) [111] and Ab [112]. It is important to consider the targeting properties of the surface ligands and the possible effects of the NPs in different parts of the body when designing therapies for biomedical applications. 

There is an extensive literature dedicated to the conjugation of chemotherapeutic agents into Au NPs via covalent conjugation or non-covalent interactions and their delivery in a targeted or non-targeted manner [113]. As an example, Gibson et al. reported the conjugation of Au NPs to the chemotherapeutic agent (i.e., paclitaxel) through a flexible hexaethylene glycol spacer using carbodiimide-based chemistry [114]. In another study, Au NPs were employed to reduce the toxicity of certain tumor necrosis factors and compared with freely administered ones, where the tumor necrosis factor alpha (TNFα) was delivered by conjugating Au NPs with a thiol derivatized PEG and the TNFα (CYT-6091, Aurimune) [115]. For example, Lee et al. proposed the conjugation of DOX loaded oligonucleotides to Au NPs as a DDS against colon cancer. In the study, the DOA (DOX-Oligomer-Au NP) formulation showed the cellular uptake by the cancer cell line with the release of the drug into the cell nucleus [116]. Another drug, methotrexate, was used for conjugation with Au NPs, showing the enhanced cytotoxicity in various tumor cell lines (e.g., human bladder cancer, human prostate cancer and human cervical cancer) compared to the free drug. When conjugated to Au NPs, the drug had a faster and higher level of accumulation in the cancer cells [117]. Besides Au NPs, the use of Au as an active thin layer coating on the surface of different types of nanomaterials such as silica NPs for cancer therapy, known also as Au nanoshells (AuNSs), was reported [118,119,120]. They are currently used in clinical trials as the silica core serves as the dielectric core while the Au shell induces the thermal ablation once the NIR light stimulates the outer Au shell electrons [121].The possibility to use Au NPs as passive agents, namely diagnostic probes, was also investigated [122,123,124]. Sokolov et al. prepared a multifunctional carrier composed of Au NPs, conjugated to an antibody to bind to the epidermal growth factor receptors (EGFR). It served for detecting cervical cancer where Au NPs acted as contrast agents for optical imaging due to their SPR scattering effect [125]. Other targets for cancer treatment include folate receptors (FRs). They are usually overexpressed in breast, kidney, brain and ovary cancer cells. By using FA or anti-folate on the Au NPs surface, they can act as targeting agents for drug delivery. Recently, Au conjugates were synthesized to target the FRs with a linker of PEG between the FA and the Au NPs [126]. However, more recently, Au NPs are also being explored as active agents with antitumor properties, i.e., theranostics. Theranostics is a new technological field which develops multifunctional nanomaterials that comprise nanosize structures, which offer the possibility to target and kill cancer cells in a regulated matter and simultaneously allow the detection of cancer cells with high sensitivity and specificity [127]. For instance, their design as tools for photothermal and imaging applications makes them promising instruments in cancer. PTT involves the absorption of NIR light by the phototransducer (e.g., Au NPs), which then converts it into heat to cause cell death at the tumor site. Lin et al. demonstrated the application of Au NPs as photothermal agents, where 30 nm Au NPs were conjugated with an Ab for a targeted therapy, where the NPs achieved membrane permeabilization and the killing of cancer cells after irradiation [128]. Kang et al. used mesenchymal stem cells (MSCs) as a component to aggregate pH-sensitive Au NPs for PTT. The Au NPs were loaded into the MSCs and were successfully delivered into the tumor tissues with an enhanced photothermal efficiency and therapeutic effect upon laser irradiation when compared to control Au NPs (non-pH sensitive) [129]. The combination of Au NPs as diagnostic and imaging probes have great potential as they can provide greater contrast in imaging techniques such as MRI, photoacoustic imaging (PAI), dark-field microscopy and X-ray computed tomography (CT), and can be used for ablation of cancer cells [130,131]. For example, Huang et al. prepared gold nanovesicles for PAI and PTT. The vesicles were based on PEG-b-PCL block copolymer with a disulfide bond at the terminus, where the Au NPs were grafted. These vesicles showed a high photothermal conversion efficiency (37%) and the simultaneous imaging in the size of the tumor-xenograft model [132]. A non-extensive list of Au NPs undergoing clinical trials and approved by European Medicines Agency (EMA) or FDA for cancer therapy is summarized in Table 2. All listed Au NPs systems involve the surface chemistry modification, along with the control of the size and shape of the NPs since these aspects will determine the enhanced biocompatibility, prevention of aggregation, interaction with the cells and the targeted transport and accumulation in the desired organs [133]. 

#### 2.2.2. Silver Nanoparticles

Silver NPs (Ag NPs) are one of the most studied and explored metallic NPs derived from noble metals, together with Au NPs for biomedical applications. Ag NPs have been shown to have authentic features and considerable potential for the development of new pharmaceutical formulations, antimicrobial agents, diagnostic and detection platforms, tissue regeneration materials and biomaterials and medical device coatings [136,137]. Ag NPs were proved to exhibit antibacterial, anti-inflammatory, antiviral and antifungal activities. In the past few decades, the use of Ag NPs in theranostics has gained considerable attention due to their unique physico-chemical properties and biological activities [138,139]. The physico-chemical properties of Ag NPs will be affected by the synthetic methods where their size can range from 1 to 100 nm [140]. The synthesis of Ag NPs includes chemical, physical and biological methods that are similar to the preparation of Au NPs [139]. The main components usually involved in the synthesis of Ag NPs are metal precursors, reducing agents and stabilizing agents. Chemical reduction, which is a fast, simple and inexpensive approach, is the most common method used for the synthesis of Ag NPs with a high yield as a stable colloidal dispersion in water or organic solvents. The mechanism of formation of the Ag NPs involves nucleation and growth of the NPs, where the reduction of Ag ions (Ag^+^) leads to the formation of the Ag atoms followed by cluster agglomeration, and finally to the formation of colloidal Ag NPs [138]. The most common reducing agents used in this method include sodium citrate, alcohol, borohydride, ascorbic acid and hydrazine [141,142]. A strong reducing agent (e.g., borohydride) can result in small particles (3–5 nm) with a fairly monodisperse distribution, while a weaker reductant (e.g., citrate) leads to bigger NPs formation (30–100 nm) with a broader size distribution [143,144,145]. It is also important to consider the capping agent for the stabilization of the NPs to avoid any agglomeration. The most commonly employed protective agents include polymers such as PEG, PVP, poly (methacrylic acid) (PMAA), polymethylmethacrylate (PMMA), CHIT, and organic compounds such as oleylamine and thiols (e.g., dodecanethiol) [146,147]. The surface charge of the Ag NPs can be controlled by a proper selection of the capping agent, which can be further used as precursor for conjugation with biomolecules. For instance, the production of Ag NPs using the chemical method was tested against a murine fibrosarcoma, where the citrate-stabilized Ag NPs were functionalized with a mouse serum albumin ligand (Ag NP-MSA). The study showed the reduction of size and delay of incidence of the fibrosarcoma when treated with Ag NP-MSA [148].

Despite all above-mentioned advantages, the chemical method very often requires the use of chemical reducing agents, which can be harmful to living organisms.

The production of Ag NPs by physical methods usually does not involve toxic chemicals and it has a fast-processing time that yields NPs with a narrow size distribution. However, agglomeration can occur as a major drawback because no stabilizing agent is used. The physical methods include arc-discharge, ball milling, laser ablation, physical vapor condensation and direct current sputtering [149,150]. Regardless of the mentioned advantages, the major drawback of this approach is its high-energy consumption; hence, the chemical method is mostly preferred. 

Last but not least, the formation of Ag NPs by biological method has emerged as the new viable option as it replaces the reducing agents and stabilizers with non-toxic molecules (e.g., antioxidants, proteins). The bio-molecules during the production of the NPs act as the reducing agent by the produced enzymes present in the system, and the Ag NPs are further stabilized by the proteins excreted through the microorganism [151]. The general mechanism starts by the entrapment of the Ag^+^ on the surface of the microorganism cells followed by the reduction of the ions, which produces the Ag nuclei to finally create the colloidal Ag NPs [152]. The use of microorganisms such as fungi, yeast and bacteria, as well as plant systems such as aloe vera, lemongrass, seaweed and mustard, as bio-reducing agents was reported for the preparation of the Ag NPs [153,154]. Biological methods have numerous advantages over the conventional one, including eco-friendliness, availability of a large pool of bio-reducing agents, a one-step process, ease of dispersion of the NPs in water and tenability of the particle size. In general, the implementation of biological methods allows for more ease of control of the size, morphology and distribution compared to the chemical methods [155,156]. For example, NPs have been prepared using *Bacillus* species, where an intracellular electron donor could exploit the bacterial metabolic process in the bio-reduction of Ag^+^ [157,158]. Due to their intrinsic cytotoxicity and physico-chemical properties, Ag NPs have been reported to induce cytotoxic effects against leukemia cells and breast cancer cells [159,160]. As Ag NPs become an interesting platform for cancer therapy, the anticancer activity of the NPs was investigated to elucidate their mechanism of action. Asharani et al. investigated the molecular mechanism and cellular effects using human brain cancer cells U251 and normal human lung cells IMR-90. The results showed that Ag NPs prepared by the chemical reduction method could adsorb cytosolic proteins on their surface; hence, they can regulate gene expression and influence intracellular factors [161]. On the other hand, several studies have shown that the generation of reactive oxygen species (ROS) can lead to serious cell damage, followed by apoptosis [162,163,164]. Overall, the proposed mechanism of action for Ag NPs to induce cell death involves activation of caspases, ROS generation, DNA damage, enhanced leakage of lactate dehydrogenase, endoplasmic reticulum stress and mitochondrial dysfunction [139]. Gurunathan et al. produced Ag NPs from *B. funiculus* bacteria and showed the effect of the NPs using different concentrations over the course of 24 h against MDA-MB 231 cells (breast cancer cells) by the ROS and activation of caspase-3 that led to the DNA fragmentation and apoptosis [159]. In addition to their innate tumor killing properties, the combination of Ag NPs with chemotherapeutic agents is an effective approach to enhance the tumor-killing effects of the drugs. For example, Zhang et al. developed Ag NPs through a biological method using a bacterium *Bacillus clausii*. The synthesized NPs had a spherical shape with a size of 16–20 nm and the combination of the Ag NPs with salinomycin (inhibitor for cancer stem cells) enhanced the cell death (81%) more efficiently than either Ag NPs (25%) or salinomycin (25%) alone against human ovarian cancer cells [18]. Similarly, Ag NPs can be conjugated with specific surface agents that are expressed in tumor cells for active targeting using different capping agents. Attaching biomolecules on the NPs surface can be achieved by physisorption or through covalent bonding based on carbodiimide (carboxyl groups) using the free amines on Ab [165]. Locatelli et al. developed a nanocarrier where lipophilic Ag NPs were entrapped into polymeric PEG-based NPs and the chlorotoxin peptide was conjugated to the surface. The results showed an enhanced cytotoxic effect and improved cellular uptake 8 times more with respect to the non-targeted NPs [166]. Recent studies demonstrated the cancer theranostics application of synthesized Ag NPs [167,168,169]. Despite all these efforts, Ag NPs are not extensively used as drug delivery agents due to serious concerns about their toxicity [137,170]. Nonetheless, as greater knowledge about in vivo behavior of the nanosystems, biodistribution, bioavailability and toxicity unfolds, so do the possibilities to apply Ag NPs as DDS; some examples can be observed in Table 3.

They demonstrated anti-tumor activity in several studies via light absorption that can promote photo-thermal elimination of cancer cells. Meanwhile, the scattered light can be used for imaging for diagnostics [160,171]. 

**Table 3:** Examples of Ag NPs as DDS.

Size of Ag NPs (nm)	Anticancer Drug	Cancer Cells	Ref.
30–50	DOX	Human lung carcinoma (A549)	[152]
		Breast cancer (MCF-7)	
32	Plumbagin	Human cervical cancer cells	[172]
23	Dexamethasone	HeLa cervical cancer cells	[173]
		Osteocytic cells (MLO-Y4)	
20	Imatinib	Breast cancer cells (MCF-7)	[174]

#### 2.2.3. Superparamagnetic Iron Oxide Nanoparticles

The concept of using magnetic materials along a magnetic field in medicine was introduced in the 1960s, and much attention has been focused on the superparamagnetic iron oxide NPs (SPIONs). They have attracted special attention as a suitable nanosystems for drug delivery (e.g., chemotherapy, gene therapy) due to their biocompatibility, ease of functionalization, higher relaxation times, inherent magnetic behavior, biocatalytic activity and photo-responsiveness. Their main advantage lies in their unique magnetic properties, which allow them to serve as contrast agents in magnetic resonance imaging (MRI) or/and as magnetic hyperthermia treatment [175]. For in vivo applications, maghemite (γ-Fe_2_O_3_) and magnetite (Fe_3_O_4_) forms of iron oxide have been commonly preferred for being metastable with a cubic inverse spinel crystal structure as compared to other forms of iron oxide. SPIONs with sizes below 25 nm exhibit superparamagnetic properties. Superparamagnetism occurs below the characteristic threshold where the SPIONs enter a single-domain state. The single-domain state refers to the state where the NPs’ magnetic moments are aligned in one direction along the respective axis of magnetization. Once the applied magnetic field is removed, the SPIONs retain no residual magnetism, hence making them perfectly suitable for magnetic hyperthermia treatment, imaging, gene therapy and tissue repair [176]. The size of the SPIONs for in vivo applications must be ideally between 10–100 nm to avoid rapid renal clearance and sequestering from the RES [177]. Additionally, the SPION’s surface must be covered by a biocompatible compound to prevent degradation of iron oxide, suppress the agglomeration of the NPs and provide the desired functional groups for bioconjugation. Various methodologies have been developed for the synthesis of SPIONs, namely co-precipitation, microemulsion, hydrothermal synthesis, thermal decomposition, sonochemical reactions, aerosol and sol-gel synthesis [178,179,180]. The co-precipitation technique is the simplest one and allows industrial upscaling for clinical applications. The size of the NPs can range from 2 to 17 nm depending on the methodology parameters. The synthesis consists of the mixture of Fe^2+^/Fe^3+^, followed by the addition of a strong base under an inert atmosphere. The formation of the SPIONs begins with a short burst of nucleation as the concentration of the species reaches supersaturation, and then a slower growth process follows by the diffusion of the solutes to the crystal’s surface. The salt precursors, pH, ratio of the salts and temperature will have an influence on the size distribution and properties of the synthesized NPs [181]. Based on the synthesis parameters for co-precipitation, attempts to prepare SPIONs using nanoreactors have been studied. For example, formation of magnetic NPs in micelles and microemulsions were produced by the oxidation of iron salts, and the size distribution was controlled by the temperature and surfactant concentration [182]. Overall, NPs with diameters from 3 to 116 nm can be obtained using the nanoreactor methodology [183,184]. For the case of hydrothermal syntheses of SPIONs, there are two main routes: hydrolysis and oxidation of the mixed metal precursors. Both processes are very similar, and again, the reaction conditions are very important for the final product properties. For example, the particle size of the SPIONs increases with a longer reaction time and a higher water content also results in the precipitation of larger SPIONs. Therefore, the particle size is mainly controlled through the rate of nucleation and the grain growth, which is dependent upon the reaction temperature [185]. In contrast, thermal decomposition occurs by the decomposition of iron organic precursors (e.g., iron pentacarbonyl (Fe(CO)_5_), iron (III) acetylacetonate (Fe(acac)_3_)) and using surfactant and organic solvents. For example, Sun et al. produced SPIONs with a diameter from 4 to 20 nm by the reaction of Fe(acac)_3_ with 1,2-hexadecanediol in the presence of oleic acid and oleylamine [186]. Even though new techniques such as thermal decomposition and hydrothermal synthesis allow the obtainment of more monodisperse distribution and enhanced magnetic properties, they often require the use of toxic chemicals for biomedical applications; therefore, co-precipitation remains the most used technique. 

**Figure 3:**
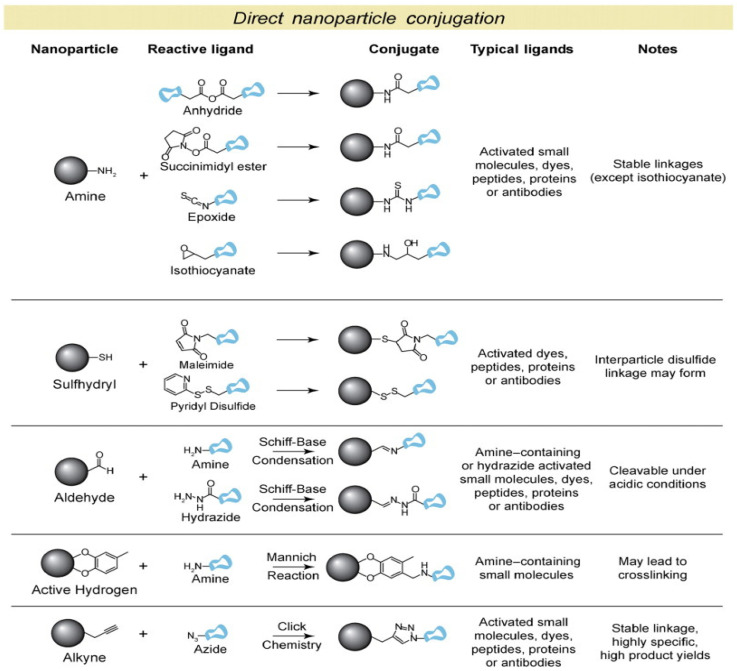

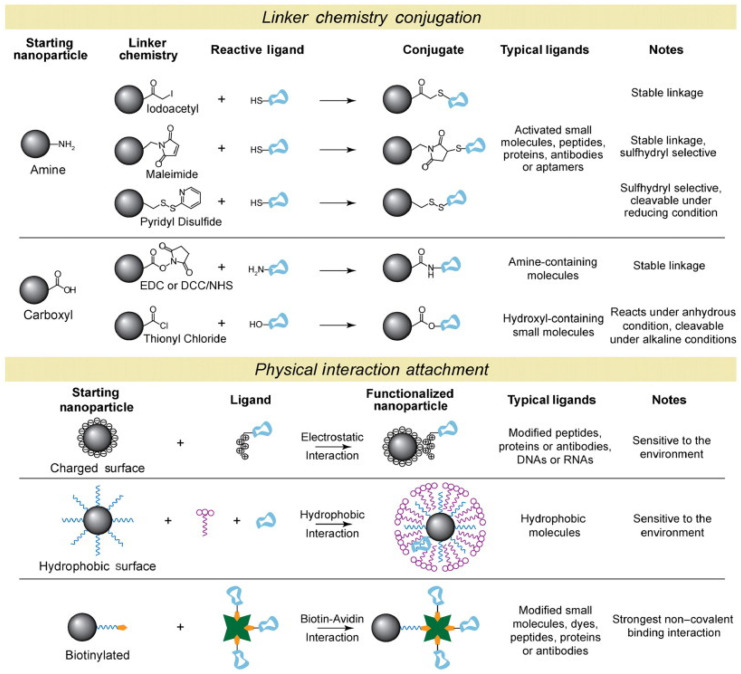
List of SPIONs surface functionalization possibilities. Reprinted with permission from ref. [179]; Veiseh et al., Adv. Drug Del. Rev. 62 (2010) 284–304. Copyright (2009) Elsevier.

The possibility to incorporate a functional group on the SPION’s surface is essential for their stabilization, cytotoxicity, target and application. So far, several studies have examined the potential cytotoxic effect of the SPIONs with different surface coatings, generally showing no or low toxicity until high exposure levels (>100 µg mL^−1^) [187,188,189]. High exposure level of SPIONs can lead to oxidative stress, DNA damage and changes in the gene expression. Therefore, is it extremely important to select a surface coating capable of stabilizing the SPIONs until they are cleared from the body [190,191,192]. First, the surface coating with desired functional groups can prevent their aggregation and cytotoxicity and can provide binding sites for further conjugation with the pharmaceutical agent or targeting ligand. Stabilization of the NPs mainly achieved by electrostatic forces or steric repulsion and by controlling the strength of these forces is the key parameter to obtain NPs with a good stability for biomedical applications. The most common primary functional groups adsorbed or attached on the SPIONs surface include carboxylic groups (e.g., alginate (ALG), polyacrylic acid (PAA), and citrate), amine groups (e.g., polyethyleneimine (PEI), CHIT) and hydroxyl groups (e.g., PEG, polysaccharides, ALG) [193]. Another way of coating SPIONs is by using inorganic materials such as Au, silica or gadolinium, which in turn can enhance the nanoparticle properties and help in binding biological ligands. Lin et al. synthesized a core-shell structure of Fe/Au NPs with a 10 nm size by a reverse-micelle approach [194]. In another example, Caro et al. synthesized Au coated SPIONs and capped with PVP (Fe@Au NPs) by a seed-mediated growth chemical method. In the study, the NPs showed a multimodal activity as contrast agents for MRI, X-ray CT and as possible image-guided PTT, thus indicating the potential development of the platform as theranostics agents [195]. Similar strategies to Au and Ag NPs are employed for the surface functionalization and stabilization of the SPIONs and are depicted in Figure 3. Overall, the conjugation efficiency will be variable depending on the surface chemistry of the NPs, and sometimes the biomolecules require modification in order to be reactive for conjugation. This is mostly the case for direct NP conjugation, and in turn loss of bioactivity can occur [196]. For example, a new approach implicates the use of “click” chemistry, which involves Copper (Cu)-catalyzed azide-alkyne chemistries [197]. Both groups are highly reactive towards each other and are unreactive with most functional groups, allowing for the specific conjugation with the desired moiety with a highly stable bond. Even though the implementation of “click” chemistry allowed for a direct conjugation of SPIONs with a biomolecule, there are some limitations. First, the Cu catalyst can lead to problems in vivo if it is not properly purified beforehand, as excessive consumption of Cu was linked to disorders such as kidney disease, hepatitis and Alzheimer’s [198]. Second, the highly stable bond between the SPIONs and biomolecules may also inhibit the further degradation of the magnetic NPs and be secreted from the body. Therefore, the method for conjugation with a biomolecule mainly involves the modification of the SPION’s surface with a proper functional group that contains a linker that can directly be attached between both moieties. The linker chemistry conjugation has a better control over the binding sites since it can increase the number of active biomolecules at the nanoparticle surface and the milder reactive conditions limit the loss of bioactivity during the conjugation. Finally, another way to achieve interactions between a biomolecule and the SPIONs is by applying physical forces based either on electrostatic interactions, hydrophobicity/hydrophilicity or affinity (e.g., avidin-biotin) interactions. The advantages when using this approach include no need for an intermediate modification step and high and rapid binding efficiencies. For example, electrostatic interactions have been used for the assembly of plasmid DNA onto SPIONs, where the SPIONs were coated with cationic PEI and then complexed with the negatively charged plasmid DNA [199,200]. While hydrophobic/hydrophilic interactions have remained unattractive for conjugation with a biomolecule due to the sensitivity of the SPIONs to the environmental conditions and low orientation of the bound ligands, affinity interactions have shown to be very effective for bioconjugation. For example, the SPION’s surface can be modified with streptavidin, which will specifically bind to any biontinylated molecules [201]. Unlike the electrostatic and hydrophobic interactions, the affinity interaction is the strongest non-covalent bond and does not suffer from environmental conditions such as salinity or changes in pH [179]. As the SPIONs systems have the possibility to be coated with diverse polymers and materials, they are capable of interacting not only with biomolecules but also with any desired drug and they can act as magnetic nanocarriers for drug delivery. The strategies for conjugation are similar to the one used for bioconjugation and can overall be represented in Figure 3.

For example, the therapeutic effect can be triggered by the applied magnetic field, or, as it is called, magnetic drug targeting (MDT). The MDT first involves the attachment of the drug to the biocompatible SPIONs, followed by intravenous injection of the SPIONs in the form of suspension and finally the application of the external magnetic field to direct the NPs towards the pathological site to allow the release of the drug. For example, early clinical trials using MDT have been reported where SPIONs were loaded with epirubicin, and the accumulation of the NPs at the target site in half of the patients was demonstrated [202]. Unfortunately, some problems are also encountered using this therapy, such as embolization of the blood vessels and control of the drug diffusion after release from the magnetic NPs, as well as some toxicity. Nonetheless, it remains as a promising new therapeutic model for cancer therapy. Another way to take advantage of the SPION’s properties as cancer therapy is by the local overheating of the cancer cells alongside the simultaneous release of the drug. This effect is called hyperthermia or magnetic fluid hyperthermia (MFH), where the SPIONs can adsorb an alternating magnetic field that is then converted into heat. The exploration of MFH began in 1957 by destroying metastases on the lymph nodes from dogs. Since then, the development of SPIONs as a potential cancer treatment has exploded. For instance, one successful outcome was developed in Berlin at Humboldt University, where the group synthesized SPIONs with an aminosilane coating against prostate cancer and glioblastoma multiform [203,204]. Due to the coating and their small size, the SPIONs could be taken up intracellularly by differential endocytosis. The testing of the new system in clinical trials resulted in a positive outcome, leading to regulatory approval in Europe. The SPION formulation (NanoTherm) is currently applied in combination with radiotherapy for patients with brain tumors [205]. Each of the proposed components can usher in endless possibilities for the assembly of the nanoclusters, which in turn needs to be designed in a clever strategy for the proper distribution within the patient. Therefore, the next section will include examples of nanoclusters with multiple functionalities.

### 2.3. Design towards Nanoclusters as Drug Delivery Systems and Theranostics

Nanoclusters have drawn considerable research interest in the biomedical field, owing to their distinct features. To effectively design nanoclusters for cancer therapy, parameters such as size, charge and surface properties must be tailored for the effective bio-distribution in patients. In this section, the design, fabrication and properties of the two main classes of nanoclusters, namely noble-metal nanoclusters and hybrid (i.e., hetero) nanoclusters and their mode of action in cancer therapy are summarized.

#### 2.3.1. Metallic Nanoclusters

**Figure 4:**
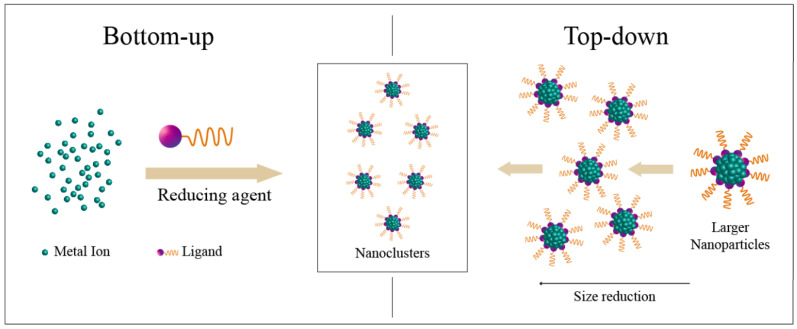
General approach for synthesis and functionalization for the metallic nanoclusters.

Metallic nanoclusters (MNCs) are nanosystems composed of a few to a hundred atoms. They display molecule-like features since their size (below 2 nm) is close to the Fermi-wavelength of electrons (c.a. 0.7 nm) [206]. Owing to their discrete energy levels, MNCs have radically different chemical, optical and electrical properties from those of bigger metal NPs. A distinct characteristic of the MNCs is their strong photoluminescence, ease of synthesis, tunable fluorescence emission, very large surface-to-volume ratios, good quantum yields, high photostability and large Stokes shift [207]. Luminescence properties of the MNCs can be controlled using several approaches, such as ligand-to-metal electron transfer, quantum confinement effects, controlled surface complexation, ligand-controlled formation of super-cluster architectures and via agglomeration induced emission between clusters protected by thiolate [208,209,210]. MNCs formed from Au and Ag have been widely explored for applications in the biomedical field for bioimaging and biolabeling [206,211]. Therefore, for the MNCs to be applied as successful tools, it is highly important to assemble a biocompatible and reliable platform to achieve the required performance. Biocompatibility is a key parameter for the short and long-term interaction with the patient, as when the MNCs are injected into the organism, they can potentially harm the host and trigger an immunological response that could lead to toxicity. To reduce and avoid the possibility of a negative response, coating and functionalization of the metallic core and coating with a non-toxic shell can improve its biocompatibility while keeping their intrinsic properties. The general approach for achieving proper functionalization for the MNCs is either by the “top-down” or “bottom-up” method (Figure 4). The most common method of synthesis is very similar to the preparation of inorganic NPs such as chemical reduction, chemical etching, photoreduction, electrochemical synthesis, sonochemical synthesis and microwave assisted synthesis [206,212]. The most widely used methodology is the chemical reduction of the metal salts in the presence of organic ligands as the stabilizing agents. To control the size of the MNCs and obtain ultra-small MNCs, three essential features need to be considered, namely a strong stabilizing effect that can be obtained by ligands with a large steric hindrance, a high metal binding affinity between the ligand and metallic core and a weak reducing power coming from the reducing agents. For instance, Au NCs can be assembled in-situ using thiols as protecting ligands. This approach consists of the chemical reduction of the Au ions into Au^0^, followed by their nucleation in the presence of capping and reducing agents (e.g., NaBH_4_ and tetrakis (hydroxymethyl) phosphonium (THP)) [213]. The array of substituents will influence the arrangement of the Au atoms and hence the NC’s core due to the volume and properties of the ligand [214]. The presence of thiols is often used because they can form strong thiol-Au bonds with a similar strength as Au-Au bonds and they have good water solubility and high stability. Many thiols have been used to prepare monolayer-stabilized Au NCs and Ag NCs, such as glutathione (GSH), thiolate cyclodextrin and tiopronin [215,216]. Besides thiols as capping agents, biomolecules and polymers have gained attention in the chemical reduction process due to their minimal toxicity and higher biocompatibility [213]. For example, several studies were performed to synthesize luminescent Ag NCs using various DNA sequences [217]. Ag NC’s functionalization using DNA helps prevent the Ag NCs from aggregating and allows a distinct recognition ability for biomarkers in the cells, e.g., integrating the DNA tail with some aptamers to attain specific binding and cell targeting [218]. The main advantages for these Au NCs and Ag NCs have been explored in the field of biosensing and biolabeling thanks to their fluorescent properties. Lately, they have gained more attention in therapeutic applications (e.g., radiotherapy and photodynamic therapy) as a new strategy to elude drug resistance [219,220,221]. For example, in radiotherapy, ionization energy is used for the killing of cancer cells. However, as a high energy is employed, healthy tissues can be also affected, which constitutes a major obstacle of this method. Therefore, the need for materials that can be localized in the tumor and be used as radiosensitizers are now being considered. Since Au has a high atomic number (Z = 79), it can have an enhanced radiation effect [222]. The use of Au NCs as radiosensitizers can be an advantage since they can strongly absorb a radiotherapy ray and yield the secondary electrons once irradiated with gamma or X-rays [223]. It was reported by Zhang et al. that Au NCs synthesized using glutathione as a coating agent can accumulate in the tumor by the EPR effect, and thus enhance the efficacy of the radiotherapy [224,225]. The potential photochemical activity of Au NCs on cancerous cells was lately explored. Cifuentes-Rius et al. synthesized protein-stabilized Au NCs as potential photodynamic agents, which displayed cell uptake even after 24 h of administration This induced cell death throughout this period via the production of ROS, including singlet oxygen, which plays a key role in the photodynamic therapy as damaging cellular factors after their irradiation with NIR light [226]. The application of Ag NCs as tools in photodynamic therapy have been also explored. For instance, in a study by Yu et.al, bovine serum albumin (BSA) protein templated Ag NCs exhibited a stronger singlet oxygen generation than their Au NCs analogue. These Ag NCs were tested on MCF-7 breast cancer cells where they showed good uptake and the cancer cells were killed by the irradiation of white light [221]. Overall, MNCs offer an excellent, versatile and multifunctional platform for developing colloidal superstructures via self-assembly governed by metal cores and surface ligands for various applications including bioimaging, therapy and drug delivery.

The cytotoxicity of the MNCs could be easier to evaluate compared to the NPs as the narrow size distribution of the MNCs will allow the oversight of the size dependent toxicity that has to be considered when dealing with NPs [227,228]. Nonetheless, the proper surface functionalization remains an important factor for their biodistribution and clearance. Among the examples that can be found in the literature [229,230,231], Zohrabi et al. prepared BSA Au NCs that were conjugated with a chimeric peptide (HNH) to decrease the potential cytotoxicity of the NCs. In this study, several ratios of HNH to BSA Au NCs were tested to find the highest transfection efficiency, showing the importance of the proper design of the platform for an optimal biosafety profile [232]. 

#### 2.3.2. Multicomponent and Multifunctional Nanoclusters

Development of nanomaterial systems featuring multiple features such as fluorescence, magnetization, drug delivery and therapy is highly desirable for biomedical diagnosis and therapy. In this regard, combination of various therapeutics or simultaneous usage of different therapeutics mechanisms within a single system would significantly enhance the treatment efficiency. During the last two decades, through the efforts of many researchers, methodologies integrating various functionalities within a single particle have been reported [233,234,235,236]. Their preparation methods can be divided into three categories [237,238,239], including a coupling method, inorganic synthesis and an encapsulation method [240,241]. Despite the multiple advantages of this approach, it frequently necessitates the usage of toxic molecules that come into contact with the biological materials, which may lead to loss of bioactivity. To address this issue, supramolecular assembly of sundry building blocks (e.g., NPs), which can be synthesized individually while avoiding handling of toxic chemicals and while manipulating biomolecules, into large hierarchical nanostructures (i.e., nanoclusters) would be more beneficial. Integration of multiple functionalities into nanoclusters by combining a variety of materials exhibiting different physico-chemical properties and functionalities has the potential to transform health care in cancer. The design and fabrication of multi-component nanoclusters with multiple functionalities (e.g., bioimaging, anti-cytotoxic activity, targeting, drug delivery and therapy) for cancer theranostics is in its infant stage. 

##### MNCs—Based Multifunctional Nanoclusters

**Figure 5:**
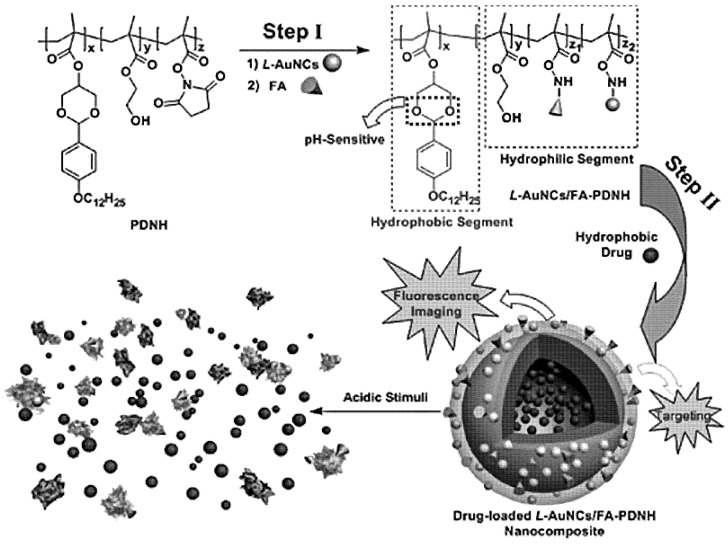
Schematic illustration of the multicomponent nanocluster system based on Au-NCs and amphiphilic copolymer. Reprinted with permission from ref. [244]; Chen et al., Adv. Funct. Mater. 23 (2013) 4324–4331. Copyright (2013) John Wiley and Sons, Inc.

Combination of MNCs with various materials (e.g., biological molecules, polymers and drugs) has lately gained significant momentum in order to construct multifunctional nanoclusters for applications in cancer nanotheranostics. These functionalities may include bio-imaging, tumor cell localization (targeting), cancer therapy and drug delivery, and can be tuned as desired since they depend on the chemical composition and the physico-chemical properties of the nanoclusters. The formation of such a multicomponent system is usually achieved by the stabilization of the MNCs with a stabilizing agent (e.g., BSA, glutathione (GSH)), followed by the conjugation to an anticancer drug, photosensitizer or a polymeric carrier or by the encapsulation of the MNCs inside the polymeric matrix [242]. For instance, Wang et al. encapsulated Ag NCs into a DNA scaffold and a nucleoli-targeting agent (AS1411) with protoporphyrin IX (PPIX) on the DNA scaffold’s surface [243]. The multicomponent nanoclusters displayed NIR fluorescence and photothermal and photodynamic efficiency against HeLa cells. Similarly, Chen et al. fabricated a pH-responsive polymeric nanocarrier encapsulating Au NCs, recognition agents and anticancer drugs to improve the selective targeting, in situ imaging and anticancer therapy against human hepatoma BEL-702 cell line and KB cell line (over-expressed folate receptor) [244]. The Au NCs were stabilized by (GSH) with the targeting ligand (FA). Then, they were attached to an amphiphilic copolymer poly (DBAM-co-NAS-co-HEMA), followed by the self-assembly of the latter with the anticancer drug (i.e., paclitaxel) (Figure 5). Both in vitro and in vivo results revealed that a multicomponent nanocluster system had a therapeutic action against both cancer cell lines (i.e., BEL-702 and KB), showing its utility for early detection and cancer therapy.

**Figure 6:**
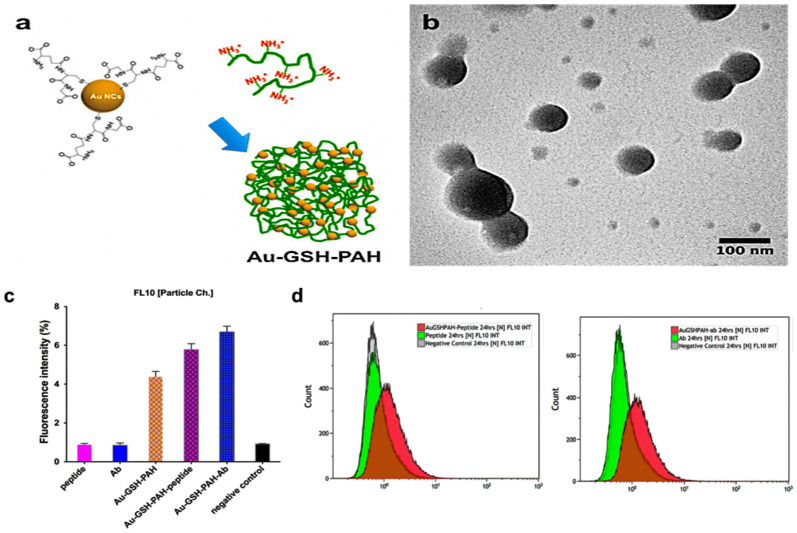
(**a**) Schematic representation of the synthesis of self-assembled Au NCs using a cationic polymer (PAH) and Au NCs stabilized by GSH, (**b**) TEM images of Au-GSH-PAH, (**c**) Detection of the fluorescence signal in cells from (**c**,**d**) the self-assembled Au NCs (Au NC fluorescence signal). Reprinted (adapted) with permission from ref [247]; Ammar et al., ACS Nano 10 (2016) 2591–2599. Copyright (2016) American Chemical Society.

In a different study, Khandelia et al. reported the preparation of Au NCs embedded with BSA (composite NPs) and the anticancer drug (DOX) [245]. The DOX loaded composite NPs demonstrated toxicity against the cancer cells (i.e., HeLa cells) while retaining their luminescence in the blood serum. Zhou et al. also synthesized Au NCs as theranostics agents while combining them with an anticancer drug (i.e., cisplatin pro drug) and FA [246]. They demonstrated the capacity of the multicomponent nanoclusters to inhibit the growth and metastasis of the breast cancer cells while exhibiting fluorescence imaging with a strong signal due to the targeting effect of the FA. Ammar et al. developed a strategy of preparation of multi-component nanoclusters based on assembly via electrostatic interactions of MNCs [247]. They synthesized Au NCs with sizes of about 100–150 nm by self-assembly. The Au NCs stabilized with GSH (Au-GSH) were used as precursor along with a cationic polymer poly (allyl amine hydrochloride) (PAH) for the self-assembly of the NPs. It was possible to form the NPs without significant aggregation thanks to a proper control of the electrostatic interactions between the Au NCs and the polyelectrolyte. The self-assembled NPs were loaded with fluorescent biomolecules (e.g., peptide or Ab) to demonstrate the drug delivery capability using a single-step reaction and determine the colocalization of the NPs and biomolecules after incubation. The loaded NPs with either peptide or Ab led to an enhancement in fluorescence as compared to their free counterparts, (Figure 6).

Incorporation of the MNCs with carbon materials has been also considered for the development of MNCs-based composites, especially, reduced graphene oxide (rGO) [248,249]. rGO displays properties such as a large surface area and hybridized carbons (sp^2^) that allow for their application in cancer therapy [250,251]. For example, Zhang et al. synthesized a carbon-based composite based on Ag NCs with an aptamer for the detection of platelet-derived growth factor (PDGF-BB), which is an important protein indicator for malignant tumors. The nanocomposite biosensor, labeled as 3D-rGO@AgNCs@Apt, exhibited biocompatibility and specificity for the protein with a low detection limit [252]. Another example includes the assembly of nanocomposites based on Au NCs, rGO and FA. The biosensor was used for the detection of two metal ions (e.g., Na^+^ and K^+^) using ribonuclease A (RNase A) that can be used as therapeutic protein. The RNaseA/AuNCs were loaded onto FA-rGO, and hence the composite can be used as a potential DDS and fluorescence quencher [253]. Another material that has been thoroughly explored is the combination of mesoporous silica NPs (MSNPs) with MNCs/inorganic NPs. The application of MSNPs in the area of nanomedicine is associated with their high surface area, chemical stability, ease of functionalization and biocompatibility [254,255]. For example, Mulikova et al. synthesized MSNPs coated with Au NPs on their surface (labeled as MS-Au NPs), hence forming nanoclusters using two types of primary particles. The prepared MS-Au NPs reached a size of 130 nm and they were tested for an effective X-ray attenuation in CT and as a DDS. The results showed an enhancement in the X-ray attenuation efficiency compared to other metal NPs [256]. Many other examples are found in the literature, where MSNPs represent a template for the assembly of multifunctional nanoclusters for cancer therapy [257,258,259,260].

##### Supramolecular Assembly of Building Blocks into Multifunctional Nanoclusters 

**Figure 7:**
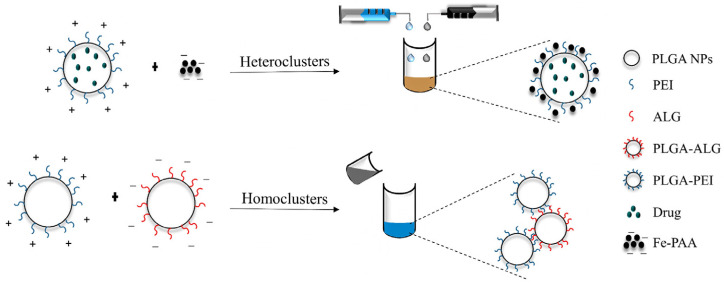
Schematic illustration of the multicomponent nanocluster system based on IO NPs and polymeric NPs. Heteroclusters assembly based on electrostatic interactions using one-shot and dropwise approach. Reprinted with permission from ref [261]; Zumaya et al., Eur. Polym. J. 133 (2020) 109795. Copyright (2020) Elsevier.

Besides the MNCs-based multicomponent and multifunctional systems, hierarchical nanostructures prepared by supramolecular assembly of building blocks (e.g., organic NPs, inorganic NPs, biomolecules, and drugs) using well-known approaches including hydrophobic interactions or electrostatic interactions of oppositely charged entities were reported in the literature. Following this concept, our group developed a simple and versatile methodology consisting of supramolecular assembly via electrostatic interactions of oppositely charged inorganic and drug loaded PNPs to construct controlled multicomponent and multifunctional nanoclusters [261,262]. Specifically, the nanoclusters were constructed by supramolecular assembly through electrostatic interactions of oppositely charged SPIONs and drug loaded PLGA NPs (Figure 7). It was demonstrated that by a dropwise approach (slow and progressive addition of one component into another), clusters in the nanometer range regardless of the ratio between the primary NPs were formed. This new methodology can be applied for the development of nanoclusters combining multiple functionalities such as imaging, targeting, therapy and drug delivery to fight cancer. Similarly, Haša et al. prepared multicomponent nanoclusters by electrostatic interactions of negatively charged liposomes, SPIONs and positively charged poly L-lysine (PLL) [263]. The control of the nanoclusters’ size and structure was dependent on the order of addition of the components. First, SPIONs were incorporated into the liposomes. Then, the SPIONs-liposomes were introduced into PLL solution. The formed nanoclusters were stabilized by adding an excess of SPIONs to block any remaining positive charge from PLL, and hence inhibit their increment in size. The liposomes clusters showed the advantage of encapsulating independently different drugs (e.g., resazurin and vitamin C) into the liposomes, while the addition of SPIONs allowed the stabilization of the final size of the clusters, and their presence can be used for a controlled release by applying a magnetic field [263].

In another example, Codari et al. produced nanoclusters made out of a statistical distribution of polymeric and inorganic particles (primary particles) using a process based on aggregation and breakup [264]. The aggregation process of the primary polymeric particles PMMA and SPIONs was approached by their aggregation into large micrometer size clusters by the addition of a salt. This was then followed by the breakage of the clusters through hydrodynamic stress in the presence of a surfactant. This presented proof of the concept can be applied to the production of multifunctional nanoclusters for biomedical applications. 

**Figure 8:**
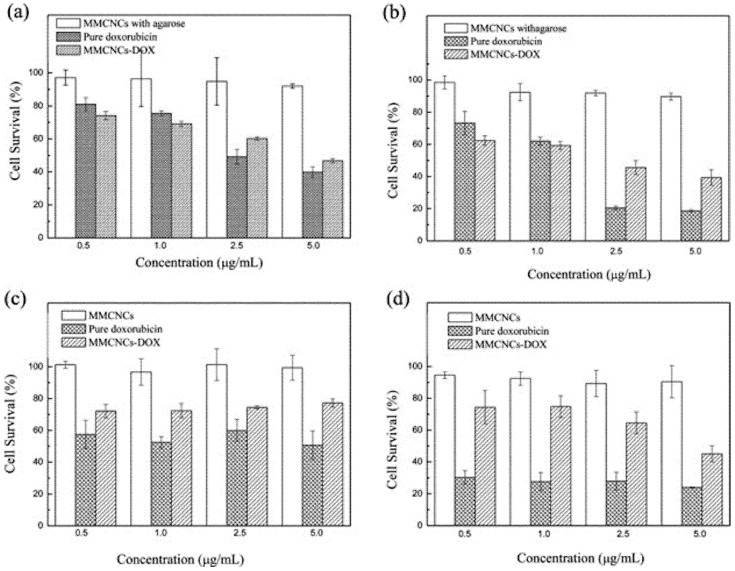
(**a**,**b**) Survival of SGC7901 cells in four different concentrations of the MMCNCs, pure DOX, and MMCNCs-DOX after incubation with cells for (**a**) 24 and (**b**) 48 h. Graphs were plotted with DOX concentrations of 0.5, 1.0, 2.5, and 5.0 μg mL^−1^. c, d) Survival of HEK 293T cells in four different concentrations of the MMCNCs, pure DOX, and MMCNCs-DOX after (**c**) 24 and (**d**) 48 h of incubation. Graphs were plotted with DOX concentrations of 0.5, 1.0, 2.5, and 5.0 μg mL^−1^. Reprinted with permission from ref [267]; Li et al., Small 8 (2012) 2690–2697, Copyright (2012) John Wiley and Sons Inc.

Thanks to their unique features, magnetic NPs (i.e., magnetic nanocrystals) of optimized size can be used as building blocks to generate magnetic colloidal nanocrystal clusters (MCNC) with tailored morphology, size and properties for anticancer theranostics applications (i.e., MRI and targeted drug delivery). One-pot as well as multiple-step solution-phase methodologies were developed to synthesize nanoscale ferrite systems consisting of several tightly coupled inorganic subunits. In the one-pot approaches (e.g., solvothermal and high-temperature organometallic methods), there is a nucleation and growth of the nanocrystals in the reaction medium. Afterwards, they agglomerate at an elevated temperature via the surfactant bridge in a secondary structure. In the multiple-steps approaches, the nanocrystals of specific shape and size are first synthesized. Then, the nanocrystals agglomerate into the nanocluster system in another synthetic step, e.g., encapsulation of the nanocrystals in organic matrixes. As an example, Xu et al. employed several biopolymers (i.e., CHIT, soybean, casein, poly (glutamic acid)) as structure directing agents for the synthesis of nanoporous MCNC by solvothermal synthesis for dual drug delivery in prostate cancer [265]. They showed that the size, crystallinity and surface properties of the nanoclusters were affected by the type of the biopolymer. The results showed the possibility of stabilizing the magnetic nanoclusters with a biopolymer and the role played by the latter in the structural changes of the porous nanocrystal clusters. Glutamic acid was chosen as the directing agent able to create a high surface area of MCNCs. Further, two drugs were encapsulated (i.e., docetaxel and ceramide) in the poly (glutamic acid) magnetic nanoclusters leading to an improvement of their drug loading and an enhancement in their apoptotic effect against PC-3 cells (prostate adenocarcinoma). The stabilization of the MNCs by a modified solvothermal approach using sodium citrate as the stabilizer was also achieved by Liu et al. [266]. The formation of magnetic NPs with a size in the range of 80–410 nm was tuned by changing the concentration of the iron salt (e.g., FeCl_3_) or sodium citrate. TEM images indicated that the obtained magnetic particles were in fact loose MNCs formed by nanocrystals with a size of 5–10 nm. The MNCs seemed to be connected to each other by the amorphous phase in the particles. The clustering of the magnetic nanocrystals provided the formed NPs with superparamagnetism and a high magnetization, making them promising candidates for theranostics. Assembly of DOX-mesoporous MNCs as a DDS was developed by Li et al. [267]. The formation of the magnetic nanoclusters were synthesized using solvothermal reaction and stabilized by agarose. Then, chemical modification of the MNCs was carried out in order to graft the anticancer drug DOX. The cytotoxicity between the free drug and the conjugated MNCs was studied in a gastric carcinoma cell line (SGC7901) and a normal cell line (HEK 293T). The results showed equivalent toxicity as compared to the free drug, but a lower cytotoxicity was present in the normal cell line (Figure 8). Dong et al. also reported the formation of highly porous magnetite clusters as a drug release system with a strong magnetic response [268]. The magnetic nanoclusters were assembled by a solvothermal reaction of the iron salt with ammonium acetate as a porogen to increase the surface area for drug loading and sodium citrate as the stabilizer and surface modifier. The overall structure was a microsphere composed of small monodispersed SPIONs with an average diameter of 150 nm, high porosity and magnetic response. The magnetic nanoclusters were monitored for drug release using a model drug (e.g., ibuprofen), showing their potential for biomedical applications.

The mentioned examples show the possibilities of assembling supramolecular structures into nanoclusters by the combination of the primary building blocks. These systems of nanoclusters can render to a higher drug loading and can implement multiple functionalities such as imaging, cell targeting and emerging cancer therapies (e.g., radiotherapy, MFH, PTT, photodynamic therapy). On top of that, the nanoclusters can be further used for drug delivery by the two main approaches: passive and active drug targeting.

## 3. Summary and Perspectives

Since the discovery of nanotechnology, the prospects of nanocarriers as DDS and theranostics have been explored for cancer therapy with an exponential rise as they offer many advantages over free drugs. They can protect the drug from degradation, enhance their biodistribution and penetration and prevent the drug from prematurely interacting with the biological environment. Although the delivery of the drugs through nanocarriers has been an alternate route, the molecular complexity of the cancer cells can present multiple drug resistance due to membrane proteins that transport the anticancer drugs out of them. Therefore, to address the challenge of multiple drug resistance and cancer recurrence, the incorporation of multiple functionalities in the form of nanoclusters has been the new approach for ensuring an efficient treatment. In fact, the preparation of the nanocarriers with tunable surface chemistry and properties has opened up possibilities for their assembly into bigger objects in the form of nanostructured materials or supramolecular systems with many functionalities such as MNCs. The combination of polymeric nanocarriers (e.g., PNPs) with inorganic NPs (e.g., Au, SPIONs, Ag) and their assembly into nanoclusters (i.e., heteroclusters) is advantageous as it offers multiple functionalities simultaneously, including imaging, targeting, therapy and drug delivery. Though less discussed in this review paper, the cited examples illustrate well the benefits of combining metallic NPs with carbon fillers (e.g., graphene derivatives) or MSNPs for the preparation of multifunctional nanoclusters as potential platforms for cancer therapy. 

Overall, it becomes clear that the exploitation of nanomaterials in practical applications holds much promise for further advances in cancer therapy. Thus, it is likely that this area of research will continue creating important advancements and results for the coming years.

## Figures and Tables

**Table 1:** Examples of polymer types designed to form PNPs for cancer diagnostics, imaging and/therapy.

Polymer	PNPs Size (nm)	Therapeutic/Ligand	Role	Cancer Type	Ref.
Polyethers (e.g., PEG-based)	40–160	Photothermal agent (IR-780), folic acid (FA)	Imaging diagnostics therapy	Ovarian, colon, breast, lung	[35,36,37,38,39]
Polyesters (PCL, PLA, PLGA and block-copolymers)	60–150	FA, doxorubicin (DOX), paclitaxel, SPION, antibodies (Ab), Indocyanine green (ICG), metal NPs		cervical cancer, HeLa cells, carcinoma (SCC7)	[40,41,42,43,44]
Polysaccharides (CHIT)	100–200			Subcutaneous tumors, prostate	[41,45,46,47]

**Table 2:** List of Au NPs for cancer therapy undergoing clinical trials and approved by EMA or FDA.

Vector	Name	Formulation	Treatment	Clinical Approve	Ref.
Au NPs	CYT-6091 (Aurimune)	PEGylated colloidal Au-TNF	Solid tumors	Phase I completed	[115]
			Non-small lung cancer	undergoing Phase II	
	AuroLase	Silica core coated with Au shell	Head and neck cancer	Completed pilot study	[134]
	NU-0129	Spherical nucleic acid formulation conjugated to Au NPs	Glioblastoma multiform	Phase 0 completed	[135]
